# Supplementary material for: Streamlining Bacillus Strain Selection Against Listeria monocytogenes Using a Fluorescence-Based Infection Assay Integrated into a Multi-Tiered Validation Pipeline
Source: Antibiotics (Basel). 2025 Jul 29;14(8):765. doi: 10.3390/antibiotics14080765 (PMC12383055; doi:10.3390/antibiotics14080765)
Supplement: Supplementary file 1 [file antibiotics-14-00765-s001.zip › Supplementary Figures v2.pdf]

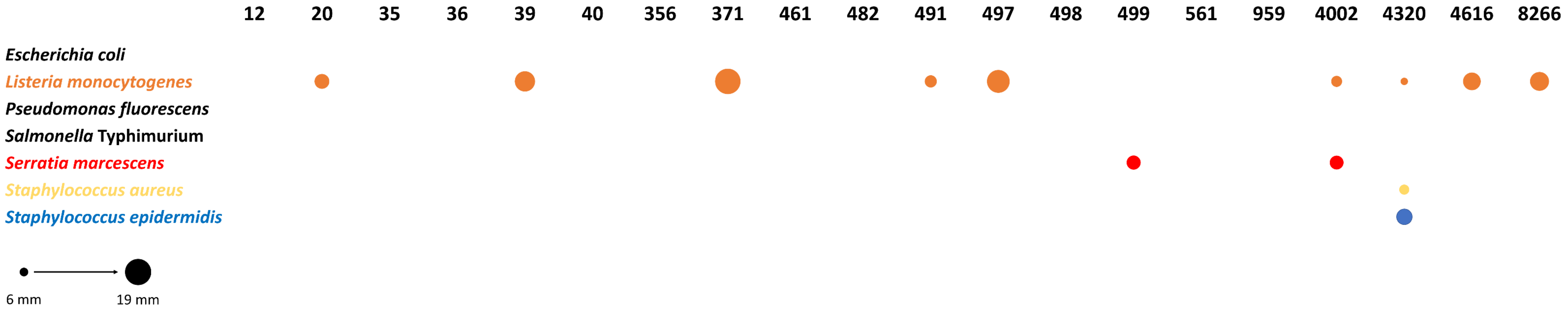

Figure S1. Bioassay results of cell-free supernatants from twenty *Bacillus* spp. strains obtained from the CECT against twelve test strains. The circle sizes are proportional to the inhibition halo diameters observed, representing the antimicrobial activity of each *Bacillus* strain.

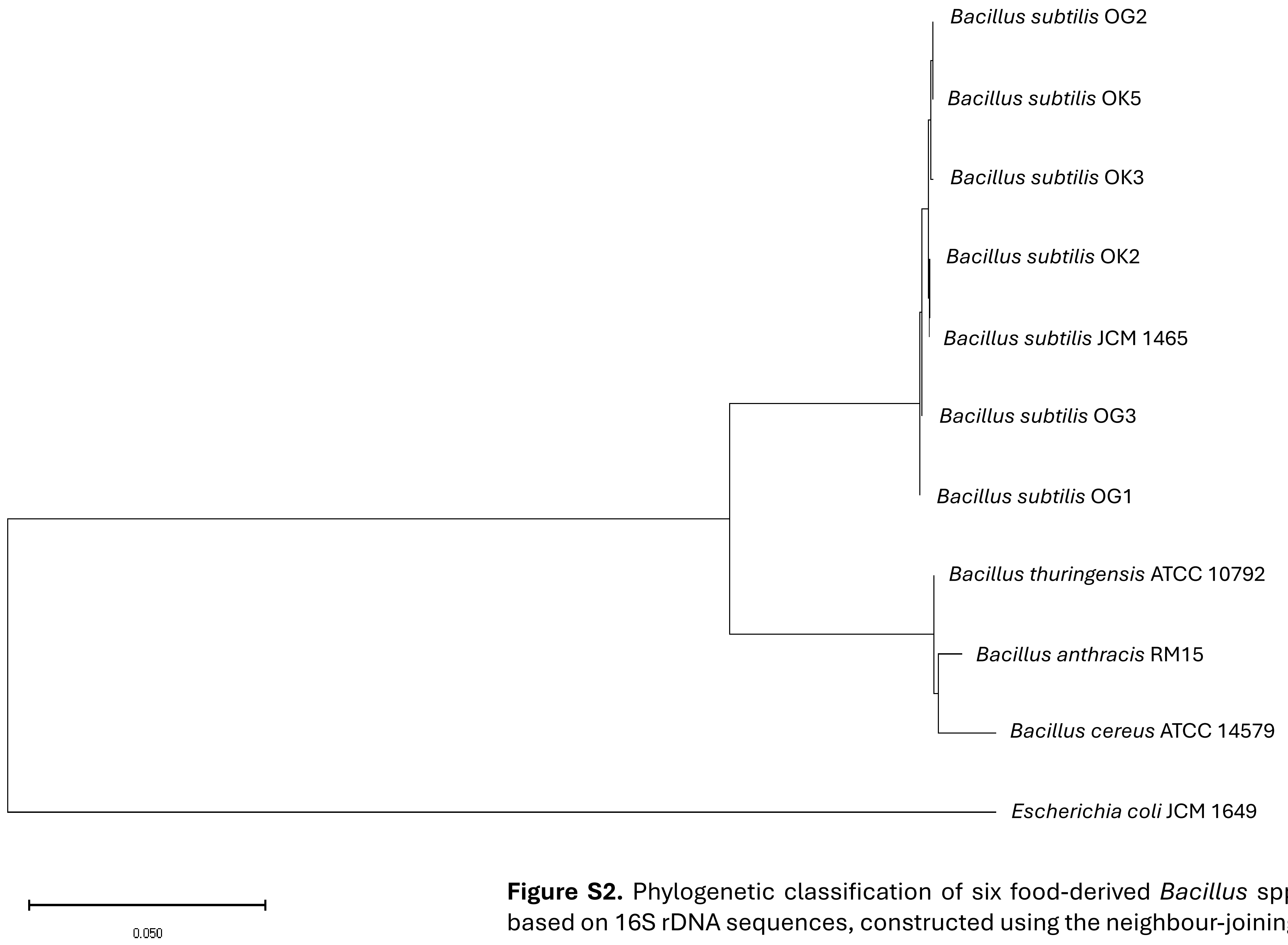

Figure S2. Phylogenetic classification of six food-derived *Bacillus* spp. isolates based on 16S rDNA sequences, constructed using the neighbour-joining method.

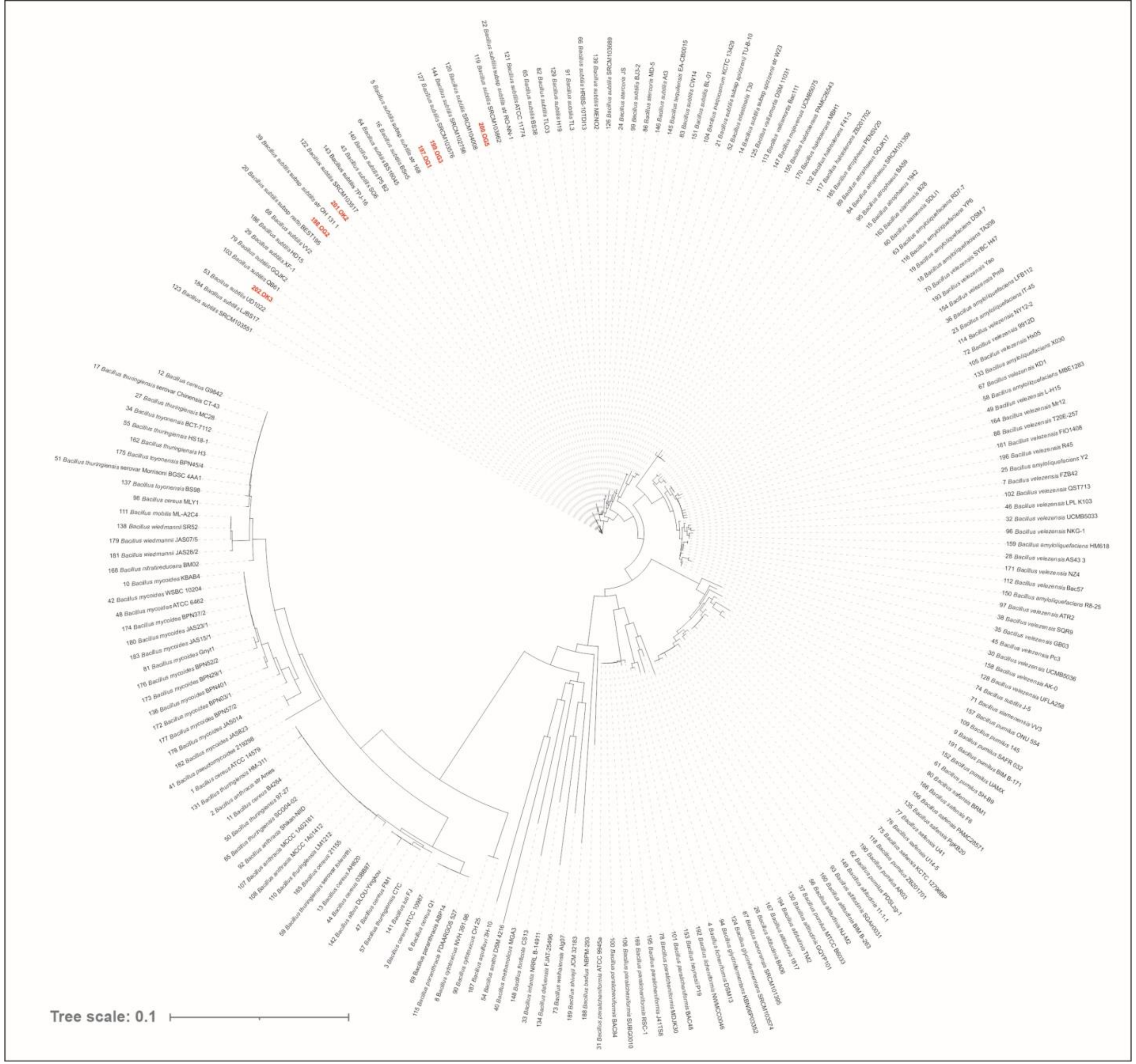

Figure S3. Phylogenetic classification of six food-derived *Bacillus* spp. isolates based on *tuf* gene sequences in comparison to over 200 *Bacillus* spp., using the neighbour-joining method.

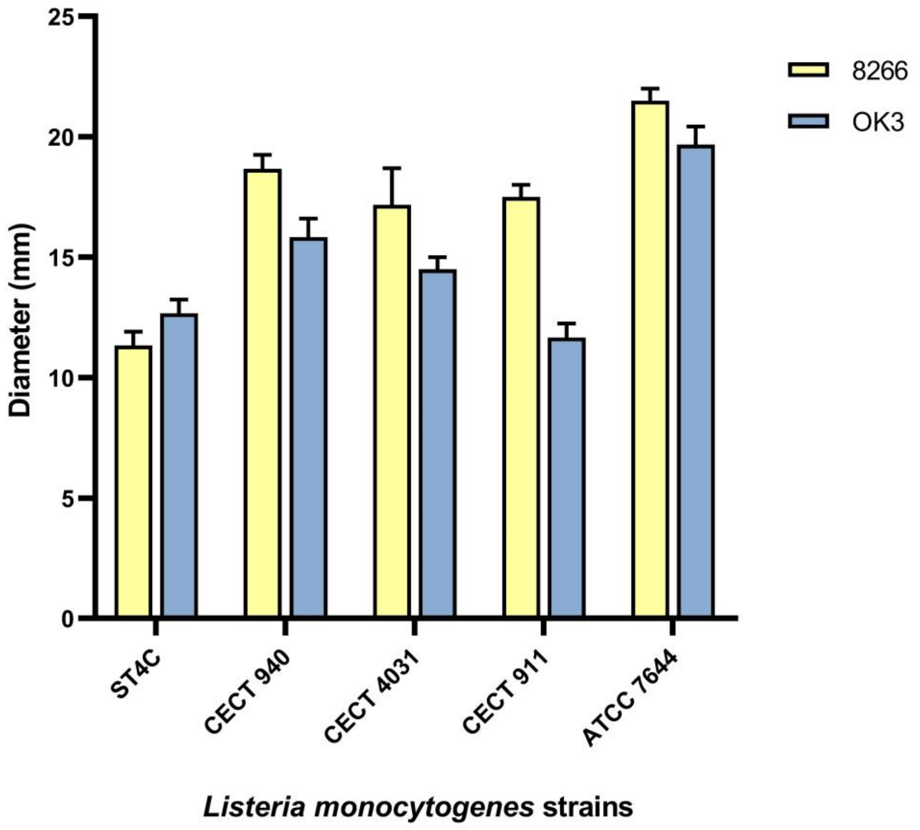

Figure S4. Antimicrobial activity of cell-free supernatants from *B. subtilis* CECT 8266 and OK3 against five *L. monocytogenes* strains, measured by agar diffusion.

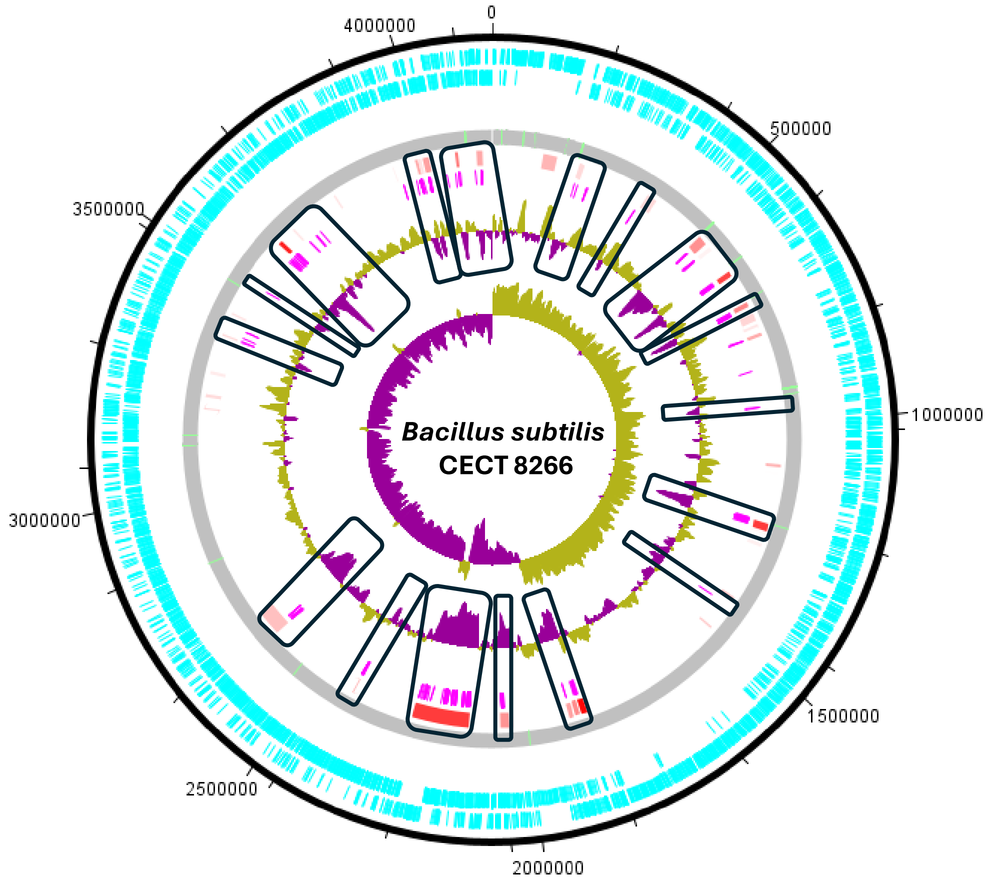

Figure S5. Circular genome map of *Bacillus subtilis* CECT 8266. The outer two rings represent coding sequences on the forward and reverse strands. The third ring marks tRNA loci, while the fourth highlights genomic regions predicted as horizontally acquired (Alien Hunter). The fifth ring displays genes unique to *B. subtilis* CECT 8266, based on BlastN comparisons with *B. subtilis* strains W32, 168, and NCIB 3610. Many unique genes co-localize with Alien Hunter-predicted regions and shifts in GC content, indicated by black rectangles. The inner plots show GC content (violet) and GC skew (yellow), with the origin of replication clearly visible.
