# Supplementary material for: Streamlining Bacillus Strain Selection Against Listeria monocytogenes Using a Fluorescence-Based Infection Assay Integrated into a Multi-Tiered Validation Pipeline
Source: Antibiotics (Basel). 2025 Jul 29;14(8):765. doi: 10.3390/antibiotics14080765 (PMC12383055; doi:10.3390/antibiotics14080765)
Supplement: Supplementary file 1 [file antibiotics-14-00765-s001.zip › Supplementary Tables v2.pdf]

SUPPLEMENTARY TABLES

Table S1. Bacterial strains used in this work.

**CECT strains / Other Colections / Genome sequence**	**Description from CECT**
Bacillus coagulans CECT 12 (Type strain) / ATCC 7050 / Genome assembly ASM83290v1	Strain derived from evaporated milk, capable of producing L(+)-lactic acid and glycoside hydrolase inhibitors. Considered a potential strain for producing single-cell protein for food or animal feed
Bacillus coagulans CECT 561 / ATCC 8038	Strain recognized for its ability to produce L(+)-lactic acid
Bacillus licheniformis CECT 20 (Type strain) / ATCC 14580 / Genome assembly ASM3447892v1	Strain known for its chitinase and chitobiase activity. Recommended by ISO standards for use in food and water microbiology, especially for assessing culture media and reagents. Additionally, its potential to promote plant growth makes it a promising candidate for use as a biofertilizer
Bacillus licheniformis CECT 959 / ATCC 25972	Strain derived from *Bacillus licheniformis* 749 due to a one-step mutation. Capable of producing penicillinase and beta-lactamase in a constitutively active form
Bacillus licheniformis CECT 4320 / ATCC 11560	Strain sourced from cassava tuber, commonly used as a quality control strain and in proteinase studies
Bacillus licheniformis CECT 4616 / LMG 7629	Strain isolated from marine environments, known to produce 2,3-butanediol
Bacillus subitilis CECT 35 / ATCC 11774	Produces penicillinase, albeit in a mild form. Frequently employed in antibiotic testing as per the European Pharmacopoeia guidelines, with additional uses in the pharmaceutical and personal care fields
Bacillus subitilis CECT 36 / ATCC 12432	Standard strain for assessing sensitivity to novobiocin (cathomycin)
Bacillus subitilis subsp. *subtilis* CECT 39 (Type strain) / ATCC 6051	Strain identified from blood samples screened for phenylketonuria, known to produce isoprene. It has further applications in testing bacterial resistance in latex paint, shows potential for single-cell protein production for food or animal feed, serves as a bacteriophage host, and is considered a potential biofertilizer
Bacillus sp. CECT 40	Isolated from environmental samples, this strain has been identified as *Bacillus cereus* through 16S rDNA gene sequencing and FAMEs profiling conducted at the CECT
Bacillus subitilis subsp. *spizizenii* CECT 356 / ATCC 6633 / Genome assembly ASM609447v1	Known for its production of subtilin, this strain is utilized in a range of applications such as antibiotic testing (including penicillin in milk and amoxicillin in blood and urine), bioautography of antibiotics, screening for phenylketonuria, sterility testing, food testing, media testing, and quality control
Bacillus subitilis CECT 371 / ATCC 9524	Strain used in antibiotic assays for penicillin, streptothricins, and streptomycin
Bacillus subitilis CECT 461 / 168t+ / Genome assembly ASM904v1	Commonly referred to as *Bacillus subitilis* 168 T+. This is a tryptophan prototrophic revertant of *Bacillus subitilis* 168, a highly characterized laboratory strain with a completely sequenced genome, heavily used in research
Bacillus subitilis subsp. spizizenii CECT 482 / DSM 618	Serves as a control strain for detecting antibiotic residues in meat from slaughtered animals
Bacillus licheniformis CECT 491 / ATCC 27811	Strain obtained from soil samples. Noted for its production of salivary and thermostable alpha-amylases. This strain is known to have a gene for maltogenic amylase
Bacillus subitilis CECT 497 / ATCC 6051a	Capable of synthesizing both alpha-amylases and proteases, this strain produces alpha-amylase (alpha-1A), salivary amylase (salivary alpha-1A), and protease (nonstructural protein 3) in substantial quantities
Bacillus subitilis CECT 498 / ATCC 21770	Retrieved from chicken and turkey manure. Known to produce alpha-amylases.
Bacillus subitilis CECT 499 / ATCC 35854	Recognized for producing both amylases and proteases, this strain produces amylase and protease (nonstructural protein 3)
Bacillus subitilis CECT 4002 / ATCC 9799	Isolated from ropy bread, this strain is notable for producing penicillinase, specifically beta-lactamase I.
Bacillus subitilis subsp. subtilis CECT 8266 / NCTC 5398	Found in a can of veal prepared for the Parry Arctic Expedition of 1820
**Food isolates**	**Description**
Bacillus subitilis OG1	Isolated from Ogiri and identified through 16S rDNA sequencing in this study
Bacillus subitilis OG2	Isolated from Ogiri and identified through 16S rDNA sequencing in this study
Bacillus subitilis OG3	Isolated from Ogiri and identified through 16S rDNA sequencing in this study
Bacillus subitilis OK2	Isolated from Okpeye and identified through 16S rDNA sequencing in this study
Bacillus subitilis OK3	Isolated from Okpeye and identified through 16S rDNA sequencing in this study
Bacillus subitilis OK5	Isolated from Okpeye and identified through 16S rDNA sequencing in this study
**Test strains**	**Description**
Escherichia coli ATCC 25922	Widely used reference strain in microbiological research and antimicrobial susceptibility testing due to its well-characterized phenotype and consistent growth behavior (Humphries et al., 2018).
Escherichia coli CECT 101	Strain considered a classic host for phages T1 through T7, obtained from CECT

Listeria monocytogenes ATCC 7644	A well-characterized reference strain widely used in research and quality control, known for its biofilm formation, stress resistance, and relevance to food safety studies.
Listeria monocytogenes CECT 911 / ATCC 19112	A serotype 1/2c strain isolated from the spinal fluid of patient with cerebrospinal meningitis
Listeria monocytogenes CECT 940 / ATCC 19117	A serotype 4d strain isolated from sheep
Listeria monocytogenes CECT 4031 / ATCC 15313	A serotype 1/2a strain isolated from rabbit
Listeria monocytogenes NCTC 7973	This strain is often studied for its virulence, resistance, and behavior in food safety contexts (Brehm et al., 1999)
Listeria monocytogenes ST4C	A poultry-derived *Listeria monocytogenes* serotype 4c isolate with strong biofilm-forming ability (Alonso-Calleja et al., 2019)
Pseudomonas fluorescens CECT 378	Strain recognized for its ability to decompose butanol, isopropanol, and methanol. It is recommended by ISO standards for performance testing of culture media and reagents used in food and water microbiology, obtained from CECT
Salmonella enterica subsp. *enterica serovar* Typhimurium SL1344	Well-characterized strain commonly used in research due to its virulence, ability to invade host cells, and suitability for studying bacterial pathogenesis, especially in the context of foodborne infections (Felgner et al., 2016)
Serratia marcescens CECT 846	Strain capable of decomposing chitin and producing various metabolites, including vitamin K and pyrazine. It synthesizes a range of enzymes, such as chitinase, L-asparaginase, and glutaminase. Additionally, it produces L-asparaginase and a follicle stimulating hormone (FSH) binding inhibitor, obtained from CECT
Staphylococcus aureus USA300	Methicillin-resistant *Staphylococcus aureus* (MRSA), known for its high-level antibiotic resistance and virulence. It is a major cause of skin and soft tissue infections, and is notable for its ability to spread rapidly in community settings, often leading to severe infections and outbreaks (Bravo-Santano et al., 2018)
Staphylococcus epidermidis CECT 231	Strain recommended by ISO standards for performance testing of culture media and reagents in food and water microbiology. It is used for a variety of tests, including antibiotic susceptibility disc testing, evaluation of antibiotics in feeds and pharmaceutical preparations, and cylinder-plate assays of antibiotics in body fluids, feeds, milk, and pharmaceutical preparations. Additionally, it demonstrates beta-hemolysis on sheep blood, obtained from CECT

Table S2. Results from anti-SMASH and BAGEL5 analyses identifying secondary metabolites produced by *B. subtilis* CECT 8266.

Region	Type	Start	End	Confidence	Most Similar Known Cluster	Cluster Type
**1**	NRPS	355,652	421,043	High	Surfactin	NRP: Lipopeptide
**2**	Terpene	1,124,694	1,145,497		-	-
**3**	transAT-PKS, PKS-like, T3PKS, NRPS	1,745,850	1,860,612	High	Bacillaene	Polyketide + NRP
**4**	NRPS, β-Lactam	1,935,683	2,013,434	High	Fengycin	NRP
**5**	Terpene	2,075,033	2,096,931		-	-
**6**	T3PKS	2,277,304	2,318,401	Low	1-Carbapen-2-em-3-carboxylic acid	Other
**7**	Terpene Precursor	2,352,270	2,373,316		-	-
**8**	Terpene Precursor	2,498,243	2,519,133		-	-
**9**	NRP-Metallophore, NRPS	3,197,891	3,249,668	High	Bacillibactin	NRP
**10**	CDPS	3,531,805	3,552,551	High	Pulcherriminic Acid	Other
**11**	Sactipeptide	3,781,141	3,802,752	High	Subtilosin A	RiPP: Thiopeptide
**12**	Other	3,806,054	3,847,472	High	Bacilysin	Other

Table S3. MICs of a selection of EFSA-recommended antibacterial compounds against different *Bacillus* spp. The data are resulting from three independent experiments with two technical replicates per assay.

	B. subtilis **CECT 39**	***B. subtilis*** **CECT 8266**	***B. subtilis*** **CECT OK3**	**EFSA's cut-off values (mg/L)***
**Chloramphenicol**	4	4	4	8
**Clindamycin**	32	4	8	4
**Erythromycin**	0.5	0.0625	32	4
**Gentamicin**	0.125	0.25	32	4
**Kanamycin**	0.5	0.25	32	8
**Streptomycin**	32	8	16	8
**Tetracycline**	4	0.125	16	8
**Vancomycin**	0.125	0.125	16	4

* Susceptible (S) ≤ x mg/L); resistant (R) > x mg/L

Table S4. CARD resistance genes in *B. subtilis* CECT 8266

Gene	Genomic Position	Mechanism	Drug Class	HGT Region
tmrB	336175–336768	Reduced permeability	Nucleoside antibiotic	No
vanT	1892049–1893233	Target alteration	Glycopeptide antibiotic (e.g. vancomycin)	Yes
vmlR	586468–588114	Target protection	Lincosamide, Streptogramin, Pleuromutilin	No
ykkC	1360422–1360760	Efflux pump	Aminoglycoside, Phenicol, Tetracycline	No
ykkD	1360760–1361077	Efflux pump	Aminoglycoside, Phenicol, Tetracycline	No
qacJ	1846782–1847469	Efflux pump	Disinfectants and antiseptics	No
qacG	3389808–3390167	Efflux pump	Disinfectants and antiseptics	No
fosBx1	1912117–1912551	Antibiotic inactivation	Phosphonic acid antibiotic	No
vanW	2036132–2037061	Target alteration	Glycopeptide antibiotic (e.g. vancomycin)	Yes
bcI	2042753–2043673	Antibiotic inactivation	Cephalosporin	Yes
vanY	2117414–2118235	Target alteration	Glycopeptide antibiotic (e.g. vancomycin)	No
aadK	2684231–2685085	Antibiotic inactivation	Aminoglycoside antibiotic (e.g. streptomycin)	Yes
